# The healthy options for nutrition environments in schools (Healthy ONES) group randomized trial: using implementation models to change nutrition policy and environments in low income schools

**DOI:** 10.1186/1479-5868-9-80

**Published:** 2012-06-27

**Authors:** Karen J Coleman, Maggie Shordon, Susan L Caparosa, Magdalena E Pomichowski, David A Dzewaltowski

**Affiliations:** 1 Department of Research and Evaluation, Southern California Permanente Medical Group, 100 S. Los Robles, 2nd Floor, Pasadena, CA, 91101, USA; 2 Department of Kinesiology, Kansas State University, Manhattan, KS, USA

## Abstract

**Background:**

The Healthy Options for Nutrition Environments in Schools (Healthy ONES) study was an evidence-based public health (EBPH) randomized group trial that adapted the Institute for Healthcare Improvement’s (IHI) rapid improvement process model to implement school nutrition policy and environmental change.

**Methods:**

A low-income school district volunteered for participation in the study. All schools in the district agreed to participate (elementary = 6, middle school = 2) and were randomly assigned within school type to intervention (n = 4) and control (n =4) conditions following a baseline environmental audit year. Intervention goals were to 1) eliminate unhealthy foods and beverages on campus, 2) develop nutrition services as the main source on campus for healthful eating (HE), and 3) promote school staff modeling of HE. Schools were followed across a baseline year and two intervention years. Longitudinal assessment of height and weight was conducted with second, third, and sixth grade children. Behavioral observation of the nutrition environment was used to index the amount of outside foods and beverages on campuses. Observations were made monthly in each targeted school environment and findings were presented as items per child per week.

**Results:**

From an eligible 827 second, third, and sixth grade students, baseline height and weight were collected for 444 second and third grade and 135 sixth grade students (51% reach). Data were available for 73% of these enrolled students at the end of three years. Intervention school outside food and beverage items per child per week decreased over time and control school outside food and beverage items increased over time. The effects were especially pronounced for unhealthy foods and beverage items. Changes in rates of obesity for intervention school (28% baseline, 27% year 1, 30% year 2) were similar to those seen for control school (22% baseline, 22% year 1, 25% year 2) children.

**Conclusions:**

Healthy ONES adaptation of IHI’s rapid improvement process provided a promising model for implementing nutrition policy and environmental changes that can be used in a variety of school settings. This approach may be especially effective in assisting schools to implement the current federally-mandated wellness policies.

## Background

Schools are an ideal setting for disseminating interventions to promote life-long healthful eating (HE) and physical activity (PA) [1]. There is no other setting where a large number of children can be provided with opportunities to regularly consume healthful meals, be physically active in recess and physical education (PE), and receive instruction in healthy living [2]. There have been a number of reviews detailing the impact of school-based interventions on HE, PA, and childhood obesity [3-5]. In general, the findings have been disappointing. However, because schools are a setting where children spend most of their time, the American Academy of Pediatrics [6] and the Institute of Medicine [7] have still recommended that changing school settings to impact child obesity as a top priorities for research.

One main reason for the lack of success in school-based interventions may be that they fail to target system-wide policy and environmental factors influencing a child’s/family’s/school’s ability to change behavior [8]. Recent studies have attempted to address this shortcoming [9-18]. Although all of these studies appeared to provide some evidence for the importance of school environmental change, they had somewhat mixed findings due to a variety of issues. These issues included 1) difficulty in implementing school nutrition environment changes (vending, cafeteria food sales, other sources of foods/beverages, etc.) due to the pressure that nutrition services faced for financial stability; 2) failure to limit unhealthy foods brought from home into a variety of school settings (classrooms, playgrounds, cafeterias); 3) lack of integration of the intervention into daily school practice because of delivery by research staff; and 4) reliance on curriculum that was difficult to implement within the context of standardized academic performance testing.

We believe that these challenges may be due in part to the implementation protocol used by the majority of previous studies. Most have used an evidence-based medicine (EBM) approach to implementation that focused on maintaining fidelity to the components of an intervention, whether it was a specific curriculum, availability and pricing of food options in cafeterias, or dissemination of marketing messages based on social change theories. Deviation from the research protocol was considered poor implementation. This EBM approach fundamentally ignores the multiple school-level variables that may affect intervention effectiveness (such as financial concerns, labor issues, staff behavior, parental reactions, etc.). Schools are community organizations that follow their own set of regulations and practices, many of which directly oppose the stringent intervention protocols required of EBM lifestyle change research [8].

An evidence-based public health approach (EBPH) may be more effective in achieving positive outcomes when trying to change school environments and policies [19,20]. The EBPH is similar to the EBM approach in that decisions are made on the basis of the best available, peer-reviewed evidence and that data and information systems are used systematically to make decisions and evaluate outcomes. However, the EBPH approach differs substantially from the EBM approach in that it relies heavily on program-planning and evaluation frameworks such as Green and Krueter’s Precede-Proceed model [21] to address the organizational level variables that may determine intervention effectiveness [22,23]. To tailor the interventions to existing organizational conditions, the EBPH approach utilizes stakeholder engagement as parts of all phases of intervention design, implementation, and evaluation [19,20].

This paper describes an application of the EBPH approach to changing public school nutrition policies and environments: the Healthy Options for Nutrition Environments in Schools (Healthy ONES) study. The Healthy ONES study was designed to address some of the limitations of previous school environment and policy interventions by adapting the EBPH Institute for Healthcare Improvement’s (IHI) rapid improvement process model [24,25] for school nutrition policy and environmental change. This model was used because it focused on how to enact organizational change by using specific implementation cycles that were designed to build capacity within the organization and sustain the changes that were made. We hypothesized that outside unhealthy foods/beverages would be significantly reduced in intervention schools as compared to control schools and as a result, obesity rates would remain constant for children in intervention schools while obesity rates for children in control schools would increase.

## Methods

### Study design

The Healthy ONES study design was modeled after the hybrid design advocated by the Veteran’s Administration Quality and Enhancement Research Initiative (VA QUERI) [24,25]. This hybrid design uses the framework of traditional randomized designs (in our case a nested cohort group randomized trial [26]) combined with formative evaluation methods that adjust the intervention based upon data collected continuously throughout the study.

Schools were followed across a baseline year and two intervention years. After the baseline year, three elementary and one middle school were randomly assigned to the intervention and three elementary and one middle school served as controls. For elementary schools, random assignment was done by matching pairs of elementary schools based upon size and location such that larger schools and schools serving similar neighborhoods were paired. Once the pair was created, one school of the pair was randomly selected to be the control school. For middle schools, there was no opportunity to match because there were only two. One school of this pair was randomly selected as the control school. The assignment of schools was done by the first author. Both the intervention and measurement of outcomes were conducted by the same people who were not blinded to condition.

Although the intervention was delivered at the school level, longitudinal assessment of height and weight for calculating academic year changes in overweight and obesity status was conducted with second, third, and sixth grade children who gave their assent and whose parents consented to this measurement. These same children were measured for three consecutive years at baseline (2008), intervention year 1 (2009), and intervention year 2 (2010). All height and weight measures were taken in the spring semester of each school year.

### Setting and participants

**Figure 1:**
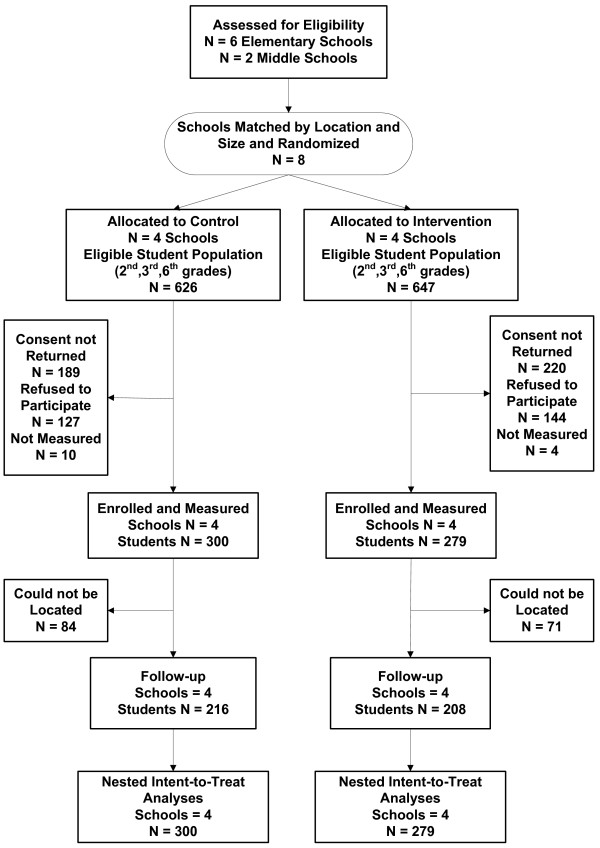
Sample selection for the Healthy Options for Nutrition Environments in Schools (Healthy ONES) study.

The targeted low income school district had six elementary and two middle schools having a total of 4,033 students, 42% Hispanic/Latino, 26% African American, 21% non-Hispanic white, and 11% other or mixed race. All children in the district were eligible for free and reduced school meals. All schools agreed to participate. Healthy ONES child recruitment, enrollment, and retention numbers are shown in Figure 1. A total of 827 second and third grade and 446 sixth grade students were eligible for the study and approached for consent. Consent forms were mailed home to parents and collected at schools. Once parents consented, assent was obtained from each child at the time of measurement.

Of the 827 consent forms distributed in elementary schools, 185 were never returned, 445 were returned with a yes response, and 197 with a no for a 69% (445/642) consent rate. In middle schools, of the 446 consent forms distributed, 230 were not returned, 135 were returned with a yes response, and 81 with a no response for a 63% (135/216) consent rate. Baseline height and weight were collected for 444 second and third grade and 135 sixth grade students.

For the same children, at the end of the first intervention year we were able to follow-up and collect height and weight from 367 second and third grade and 113 sixth grade students. Finally, at the end of the second intervention year for the same children we were able to follow-up and measure 325 second and third and 99 sixth grade students for a final retention rate of 73%. All children lost to follow-up were because they had moved out of the school district. The intervention reach was 51% (424/827).

### Intervention model

**Figure 2:**
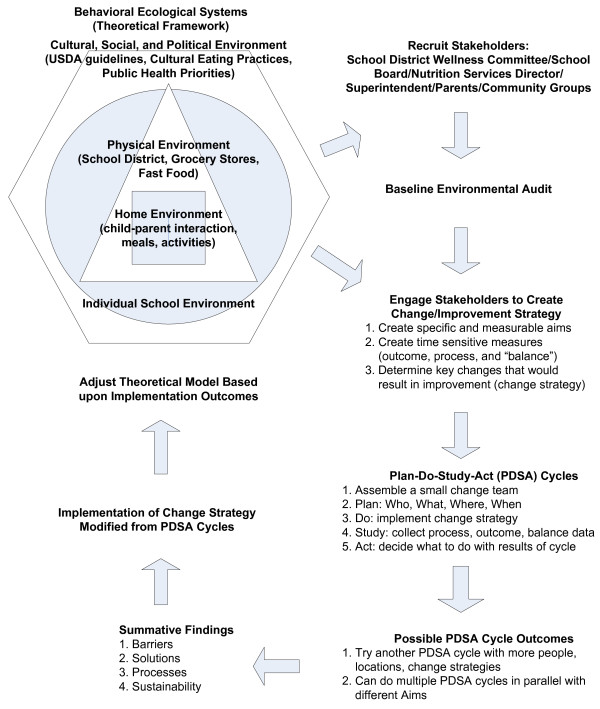
Healthy Options for Nutrition Environments in Schools (Healthy ONES) intervention model and implementation protocol.

Figure 2 outlines the elements of the Healthy ONES EBPH model. These elements were based on processes developed by the IHI for rapid improvement in health care systems [24,25], implementation research [22,23], and community-based participatory research principles (CBPR) [27,28]. The intervention had specific goals/hypotheses (i.e. eliminating unhealthy foods/beverages from school campuses – see Table 1) grounded in behavior and systems change theories [29-31], however, the strategies for change were developed by implementing the IHI rapid improvement process model pioneered in public health and large clinical care institutions such as the VA [22-25].

**Table 1:** Healthy ONES efficacy study intervention goals, target environments and strategies

**Target Environment**	**Target Strategy**
***Goal: Eliminate unhealthy foods and beverages on campus.***	
**Classroom**	·Exclusive use of nonfood rewards
	·Unhealthy foods and beverages from home not allowed
	·Fundraising with nonfood activities
**Before/After School**	·Unhealthy foods and beverages from home not allowed
	·School-wide fundraising with nonfood activities
**Recess**	·Unhealthy foods and beverages from home not allowed
**Cafeteria (school meals)**	·Unhealthy foods and beverages from home not allowed
	·Stop sale of competitive foods during school meals
	·Advertising/Mmarketing of approved healthy foods and beverages only
***Goal: Develop nutrition services as main source for healthful eating (HE).***	
Classroom	·Provide active nutrition education (i.e. field trips to cafeteria)
Before/After School	·Communicate nutrition messages to parents
	·Provide only healthy foods/beverages
Cafeteria (school meals)	·Advertising/marketing of approved healthy foods and beverages only
	·Communicate nutrition messages to parents
	·Sales of approved healthy foods and beverages
***Goal: School staff modeling healthful eating (HE).***	
Classroom	·Teachers not consuming unhealthy foods/beverages
	·Teachers promoting HE messages
	·Reward teachers for HE activities
Before/After School	·School staff not consuming unhealthy foods/beverages
	·Reward school staff for HE activities
Recess	·Teachers not consuming unhealthy foods/beverages
	·Reward school staff for HE activities
Cafeteria (school meals)	·School staff not consuming unhealthy foods/beverages
	·Reward school staff for HE activities

In 1995, the IHI developed the rapid improvement process to accelerate healthcare improvement through collaborative learning. The founders of the rapid improvement process felt that system-wide change could only happen if the process of change was conceptualized differently. Most healthcare initiatives were mandated by clinical leadership based upon efficacy studies tested in controlled, homogenous clinical trials (i.e. the EBM approach). There was no effort to involve the physicians and staff who would implement the initiative nor was there any effort to test and adapt the clinical trials protocols to “real-world” clinical settings. Consequently, systems remained static and healthcare innovations rarely made it into daily clinical practice. The rapid improvement process has now been applied in hundreds of settings across the U.S. and has resulted in the improvement of a number of chronic disease outcomes such as diabetes and congestive heart failure [24,25].

There are several elements from the rapid improvement process that are uniquely suited to address the gaps in changing obesogenic policies and environments in school settings: 1) capacity building within the organization to adapt evidence-based interventions to address organizational concerns (rather than relying on research staff to implement); 2) fostering ownership and sustainability as part of every step in the process of change; 3) providing several temporary Plan-Do-Study-Act (PDSA) learning cycles for people to “try out” change without committing large amounts of organizational resources for long periods of time; and 4) allowing for incremental and dynamic change in that goals are revisited depending upon the feedback from the PDSA learning cycles.

Another added advantage of this implementation approach is that data collection is essential to the process of change, helping the organization see the value of research as an inherent activity for capacity building. The research team becomes a partner in organizational development rather than one more outside force to contend with when trying to make decisions about policy and environmental change. Researchers are seen as community assets to achieve the goal of improving school health.

### The Healthy ONES intervention protocol

The steps for the implementation protocol are shown in Figure 2. Rather than use a purely participatory approach as is recommended in CBPR, [27,28] we used the VA QUERI model’s approach of having the research team provide guidance in EBPH approaches to the stakeholders [22,23]. This meant that we began the first step in the implementation protocol (recruiting stakeholders) by presenting them with a tentative plan (shown in Table 1) for targeted policies, environments, and behaviors.

Elements in Table 1 were chosen based upon the available evidence-based strategies from other school-based environment and policy interventions [9-18,32], ecological and developmental systems theories [29,30], behavioral ecological models [31], and the experience of the research team working in public school settings to promote PA and HE over the past 15 years [9,33-35]. Before funding was obtained for the project, preliminary support was secured for this plan from the district supervisor and nutrition services director.

### Step 1: Recruit stakeholders

The first step in the protocol began in the first year of the project (baseline) and required an advisory board be formed at the district level. The existing district wellness committee served as this overall advisory board for Healthy ONES which contained parents and the nutrition services coordinator. As part of the PDSA learning cycles (described later), change teams were required at each intervention school. Depending upon the targeted change, members included front office staff, custodians, lunchroom and recess monitors, cafeteria managers and their staff, teachers by grade, a variety of parent interest groups, and principals and vice principals.

### Step 2: Gauge organizational readiness/conduct environmental audit

In this step, traditional EBPH and CBPR approaches have the organization conduct a self-assessment to understand the general priorities of the district and staff, assess readiness to make changes, and assess barriers that might exist in making these changes. However, past research has documented that district and school administrators do not want to allocate the time to do these activities [36]. In addition, these assessments have been based upon self-report and there is now substantial evidence that self-report (whether individual or for system change) does not reflect objectively monitored practices [37]. Without an understanding of *actual* practices that exist within the community and school it is very difficult to address the initial conditions to both intervene effectively and measure the impact of that intervention.

To address these issues, the Healthy ONES protocol included semi-structured interviews with school district administrators (district supervisor and district nutrition services director) and school principals to gauge their reactions to the intervention plan shown in Table 1. We also gathered documents such as the wellness policy and any other health-related policies created by the district. Finally, Healthy ONES utilized behavioral observation to gather objective information about nutrition practices in each school. Behavioral observation was used to provide the same information as self-assessments utilized in CBPR without the inherent response demands that exist when asking people about their own or their organizations’ healthy policies and practices.

### Step 3: Engage stakeholders to create strategy for change

This step occurred at the end of the baseline year, after randomly assigning schools to intervention and control groups. The Healthy ONES District Advisory Board, recruited in step 1 of the protocol, used the findings of the environmental audit in combination with the proposed intervention plan (see Table 1) to begin the IHI rapid improvement process. This began with creating an overall change strategy which included 1) specific and measurable aims/objectives, 2) time sensitive measures of outcomes, process, and unintended consequences, and 3) an initial set of evidence-based key changes that were most likely to result in improvement. To implement each of these key changes in policy and behavior we used the IHI PDSA learning cycles protocol.

### Step 4: PDSA learning cycles

The PDSA learning cycles were used as the intervention *implementation* protocol. This protocol was standardized across all schools. The first step in the protocol was to put together a small change team at each school (3 – 4 people) who were key stakeholders in the behavior or policy to be changed and then the Healthy ONES intervention team guided them through the process of deciding who would implement the change, what would change, where the change would take place, and the timeline for implementing the change. The second step was to actually implement the change and the third step was to study the change using data collected about the process, outcome, and unintended consequences specific to this particular change. Finally (step four), the results were compiled and the change team evaluated the outcomes and decided the next steps with the guidance of the Healthy ONES intervention team.

In typical PDSA learning cycles, the outcomes are usually subsequent cycles that add more locations, more participants, and different change strategies [24,25]. Often, multiple behaviors or policies targeted for change are done in parallel, although this was not done in Healthy ONES because of the limited number of research staff to assist school staff with implementation. For Healthy ONES, each of the target behaviors in Table 1 were subjected to a series of PDSA learning cycles one at a time and most started with a single school, followed by modifications and continued learning cycles at the same school, progressing to other schools for the final set of cycles.

The PDSA learning cycles were very similar to pilot testing in research. The changes in each cycle were small, easily managed, and measured in a short period of time. Once change strategies were subjected to the cycles, summative findings were prepared for each change strategy with successes, barriers, and sustainable solutions. Once the summative process was completed, successful change strategies were implemented in all intervention schools.

### Steps 1 – 4: Intervention protocol implementation

The resulting intervention changes from the implementation of the Healthy ONES intervention protocol are summarized in Table 2. A detailed discussion for each activity shown in Table 2 in school is beyond the scope of this paper. As an illustration, we present one example of how the steps of the Healthy ONES intervention protocol resulted in nutrition policy and environmental change. This example was implemented to address the intervention goal of “eliminate unhealthy foods and beverages on campus”.

**Table 2:** Healthy ONES changes after implementation of the Institute for Healthcare Improvements(IHI) rapid improvement process

**Target Environment**	**Target Strategy**
***Goal: Eliminate unhealthy foods and beverages on campus.***	
Classroom	·Treasure chests filled with nonfood rewards for 4th and 5th grades
	·Unhealthy snacks brought from home discouraged by teachers
	·Healthy food/beverages and nonfood items for classroom celebrations
	·Nutrition Services catered meals for classroom parties
Before/After School	·Created healthier menu for after school snack
	·Changed PTA fundraising to include nonfood events i.e. Jog-A-Thons
	·Traditional carnival activities became healthy i.e. cake walk to prize walk
	·Removed unhealthy foods from PTA sponsored event menus i.e. nachos, candy
	·Added fruits, vegetables and complete meal options to PTA event menus
	·Partnered PTA with Nutrition Services to cater healthy foods for events
Recess	·Implemented daily fruit at recess program
	·Staff proactively discouraging students from consuming unhealthy snacks during recess
	·“Healthy & Unhealthy” snack poster displayed for ease of snack coaching at recess
Cafeteria (school meals)	·Cafeteria monitors proactively discouraging unhealthy food/beverages from home
	·“Healthy & Unhealthy” snack poster displayed for ease of snack coaching at lunch
	·Removed perceived unhealthy items from menu i.e., nachos, cinnamon bun, chocolate milk
	·Exclusive use of nonfood rewards by custodian and cafeteria staff for student helpers
	·Added healthier, in-house prepared entrées to menus
***Goal: Develop nutrition services as main source for healthful eating (HE).***	
Classroom	·Catered healthy meals for classroom celebrations
	·Include nonfood item as part of meal for extra celebration
Before/After School	·Created healthier menu for after school snacks
	·Catered healthy menu items for after school events and celebration
	·Supported student chef clubs/student cooking classes
Recess	·Provide cut fresh fruit at recess
Cafeteria (school meals)	·Increased student ability to consume fresh fruits and vegetables
	·Advertising/marketing of approved healthy snack and beverages only
	·Student taste tests of new menu items
	·Free meal for staff who eat school lunches with students
	·Encouraged parents to try meals to demonstrate they were healthful and flavorful
***Goal: School staff modeling healthful eating (HE)***	
Classroom	·Teachers promoting HE messages in classroom
	·Teachers proactively discouraging students from bringing unhealthy snacks to school
	·Teachers informing parents of school healthy celebration and snack policy
Before/After School	·Staff not consuming unhealthy food and beverages in front of students
	·Staff participating in parent nutrition meetings
	·Staff participating in student chef clubs/cooking classes
Recess	·Staff provided with thermal mugs to conceal caffeinated beverage consumption
	·Staff participates with students in the fruit at recess program
Cafeteria (school meals)	·Staff choosing to eat the school lunch
	·Staff encouraging their students to eat/try fruits and vegetables
	·Staff supporting nutrition services changes by encouraging children to eat school meals

One of the target environments for Healthy ONES was school-wide events which included the before and after school setting especially with respect to school fundraisers. During the environmental audit (step two in the protocol outlined previously) it was clear that school-wide fundraisers were all food-related and almost always provided high calorie, low nutrient foods and beverages such as baked goods, sugar-sweetened beverages, and high fat items like nachos. After schools were randomly assigned to intervention and control conditions, the Healthy ONES intervention team met with the district advisory board to discuss these findings. In this meeting we created specific and measurable aims to change these fundraising practices and determined the key changes that would result in improvement (step three in the protocol). Unlike other strategies we used which were controlled by district nutrition services, fundraising was very particular to each school setting. It became clear that we would not be able to work with our district advisory board to fully implement step three of the intervention protocol and would have to shift this work to the change team at an intervention school assembled for the first PDSA learning cycle. Thus we began to determine which stakeholder groups at each intervention school would be best suited to address the fundraising activities. As part of this work, the intervention team educated different groups about the district wellness policy and the Healthy ONES intervention goals. Through these interactions, one intervention school had greater readiness to change the fundraising activities.

For the first PDSA learning cycle, the change team was made up of the school principal and the parent teacher association (PTA) members who were organizing a fall carnival at this school. The plan was to change the “cake walk” to a “prize walk” by substituting half of the cakes for nonfood prizes. Nonfood prizes were provided by the Healthy ONES intervention team. In addition, the local change team agreed to offer healthier food items for the dinner portion of the fundraiser including vegetarian/cheese pizza, fresh fruit, and a mixed green salad. Parents would oversee the process and the Healthy ONES intervention team observed and recorded the choices made by families throughout the evening. The PTA also agreed to provide the fundraising results to the intervention team and compare these to the previous year’s earnings for the same event.

Following the first PDSA cycle at the first intervention school, information was summarized and presented to the local change team for their feedback. Observations documented that when children were allowed to select an item from the “walk” they always selected the nonfood prizes. When parents selected items they always selected cakes. Because more children selected items than parents, the nonfood prizes were all selected by the end of the event with several cake prizes remaining. In addition, the PTA made as much money as they had in previous years with unhealthier choices. Although there were some complaints from adults about the healthier and nonfood alternatives at the carnival, there were as many positive comments about having healthier options. Most importantly, complaints did not affect the amount of money that was raised. An unanticipated positive outcome for this cycle was that school nutrition services prepared the healthy dinner options for the event. This addressed another intervention goal, “develop nutrition services as main source for healthful eating (HE)” even though this was not the target goal for this particular intervention activity. Another unintended positive consequence was that the money spent on food for these events came back to the school (nutrition services) instead of being given to outside vendors.

Following the completion of the first PDSA cycle, the Healthy ONES intervention team disseminated the summary findings to the other intervention school parent groups. This began a phase of second PDSA cycles that occurred at all intervention schools. For the first intervention school, their second PDSA cycle focused on adding nutrition services to their change team and planning to have nutrition services provide only healthy food alternatives for all school-wide events that involved parents, teachers, and students. The Healthy ONES intervention team assisted the change team in documenting the outcomes for this second PDSA cycle by collecting opinions from teachers, parents, and students about the changes made to their events. Funds raised at any of these events through the sale of tickets for the meals were also summarized and at no time did the school make less money than in previous years with unhealthier fundraisers.

For the other intervention schools, the second PDSA cycle used the same implementation protocol (creating a change team and a plan, implement and study the change, and then decide what the next steps should be) to have fall and spring carnivals with 100% prize walks and healthy alternatives for foods and beverages. Change teams always contained teachers, parents, and nutrition services. At times children were also involved by working with teachers to implement the changes. Healthy ONES intervention staff informally observed the process, gathered financial data, and then provided the information in summary format for the change teams. These intervention schools then moved on to a third set of PDSA cycles to implement the healthy foods and beverages at all school-based events like the first school did in its second PDSA cycle.

At the end of the first year of the study, the Healthy ONES intervention team summarized the findings from all the PDSA cycles for the district advisory board as well as the other key stakeholders at each intervention school. A number of findings were shared with these groups about strategies for changing fundraising to meet the intervention goals. For example, comparisons of sales of vegetarian/cheese pizza, fruit, and salad sold separately with these items sold as a healthy combination meal found that the combination sales were better and more fruit and vegetables were consumed with the healthy combination. In addition, a “Jog-A-Thon” event was substituted for a food-related fundraising event at one school, based upon information regarding alternative fundraising practices provided by the Healthy ONES intervention team, and the revenue was greater with the Jog-A-Thon. Based upon these findings, the district supported a healthy fundraising guideline that provided information about how to have healthy meetings and fundraisers in the second intervention year.

### Outcome measures

#### Height and weight

Children removed their shoes and any heavy clothing before having their weight measured to the nearest 0.25 lb on a standard balance scale. Height was measured to the nearest 0.25 in using a stadiometer and body mass index (BMI) was calculated by dividing weight (kg) by height squared (m^2^). Data were converted to BMI Z scores and percentile BMI values. Z scores have been recommended for assessing parametric changes in children’s classification as overweight or obese instead of simply using BMI or body weight [38]. Overweight or obese was defined as ≥ 85^th^ percentile BMI and obesity as ≥ 95^th^ percentile for age and gender using the Center for Disease Control and Prevention (CDC) growth charts.

#### Behavioral observation

Behavioral observation of the nutrition environment was used to index the amount of outside foods and beverages on campuses and serve as an objective indicator of the changes in policies and organizational behaviors required of the Healthy ONES intervention. Three observation systems were developed for the four main nutrition-related organizational behaviors/environments targeted by the intervention (see Tables 1 and 2): school lunch/cafeteria, morning snack recess/playgrounds in elementary schools, and classrooms/school-wide events. These systems were developed using protocols developed for physical activity, [39] nutrition environments in restaurants and grocery stores, [40,41] and basic principles in behavioral observation [42].

For both the lunch/cafeteria and morning snack recess/playgrounds environments the observations were made based upon what children were consuming during the observation period. For the classrooms/school-wide events we were not allowed to observe inside classrooms and it was too difficult to reliably quantify items during school-wide events, thus data were collected through systematic observation of school trash. All detailed protocols are available upon request.

After development and testing of each system, training and reliability testing were conducted. Training consisted of the first author and all observers conducting observations and discussing all findings as observations were being made. Adjustments were made to the protocol when procedures were not clear. Following training, a final observation session was conducted with each system to calculate inter-rater reliability. Each time a new observer was added to the research team, they underwent the same training and reliability procedure. An equal number of observations were made across both control and intervention schools approximately once per month throughout the school year and at holidays such as Halloween, Thanksgiving, Christmas, and Valentine’s Day.

Across observation tools, inter-rater reliability for total number of items recorded per observation ranged from r = .78 to r = .99. Test-retest reliability for the total number of observed items in control schools using the classroom/school-wide events system was r = .86 (fall semesters) and r = .81 (spring semesters); r = .90 (both fall and spring semesters) for the morning snack recess/playgrounds system; and r = .90 (fall semesters) and r = .98 (spring semesters) for the school lunch/cafeteria system.

### Sample size

To calculate sample size per school we estimated a small effect size (d’ = 0.1) for the interaction of group by time based upon our previous school health work [35]. Sample size for this interaction was estimated at 50 children per school to achieve a power = .88 at an alpha level of .05. The intraclass correlation for the nesting of children in schools was also estimated to be small at r = .01. The variance inflation factor was estimated as [1+ (m-1)*r = 1.49], [43] where m = 50 was the average cluster size. The total sample size was thus estimated at 75 children per school for the three year study design (n = 450).

### Analyses

For the height and weight data, intent to treat multilevel models were used to determine main effects and interactions among different settings (i.e. schools) and individual characteristics. To adjust for the clustered data structure and determine the impact of the primary outcome measures, a mixed model analysis of covariance (ANCOVA) was conducted for the impact of the intervention on the rates of obesity over time. The main effects and interactions for the following fixed factors were included in the model: intervention (control, intervention), gender (boy, girl), and year (baseline, year 1, year 2). The following variables were considered random effects in the model: school, student, time nested within school, and the error associated with repeated measures. Baseline data were treated as a covariate in the model. Data were analyzed with all participants in the model regardless of whether or not they had follow-up measures and again using only the participants for whom we had all three measurement points. Data are presented for all participants regardless of whether or not they had follow-up. There was no difference in outcome between the two analyses. Percentile outcomes (obese yes/no; overweight or obese yes/no) were analyzed using Proc GLIMMIX (SAS version 9.2, SAS Institute Inc., Cary, NC).

Behavioral observation outcomes were analyzed using a mixed ANOVA with one repeated measure of time and two between subjects measures of intervention and environment (recess, lunch, classroom). These data were aggregated across schools thus it was not necessary to control for the nesting effect of students in schools across time. In order to compare schools and to examine changes over time, total item counts across environments were corrected for the size of each school. Larger schools had greater amounts of items simply because they had more students and staff. This was calculated by either counting the number of students in the environment during observation (school lunch/cafeteria and morning snack recess/playgrounds observations) or obtaining the student attendance for the day of observation (classroom/school-wide events). Using this adjustment, outcome variables used for the observational analyses were items per child per week.

To further understand the impact of the intervention on the types of outside foods and beverages brought to campus, we created four broad categories: Unhealthy foods, unhealthy beverages, healthy foods, and healthy beverages. These broad categories were created using the standards from California Senate Bill 12, [44] the United States Department of Agriculture HealthierUS School Challenge, [45] and the Institute of Medicine [46] guidelines. A healthy food had to have *all* of the following characteristics: total calories ≤ 173, total fat content ≤ 35% of total calories, total saturated fat content of ≤ 10% of total calories, sugar ≤ 15 g, sodium < 200 mg, and trans fat ≤ 0.5 g. Only 100% juice or water, unflavored nonfat, 1%, or 2% milk, and soy or rice milk were considered healthy beverages.

## Results

### Participants

Children were 57% girls, 8.9 ± 1.6 years old, with an average weight of 36.8 ± 12.4 kg, and BMI of 19.36 ± 4.12 kg/m^2^. Forty-three percent were overweight or obese and 25% were obese with an average BMI Z score of 0.77 ± 1.06. The racial/ethnic distribution of the study sample was 52% Hispanic, 19% African American, 19% non-Hispanic white, 7% Asian/Pacific Islander, 0.3% Native American, and 2.7% unknown. Compared to the overall district population, the study sample had more Hispanics (42% district) and fewer African American (26% district) and non-Hispanic white (21% district) children. There were no differences in rates of obesity, rates of overweight, age, or gender between control and intervention group children at baseline. However, intervention group children had higher BMI Z scores (0.86 ± 1.03) at baseline than control group children [0.68 ± 1.10; t(577) = 2.06; p = .04].

There were no differences in rates of obesity, age, and gender at baseline between children who had measures for all time points (n = 424) as compared to those who did not (n = 155). However, children who had measures for all time points had significantly *higher* BMI Z scores [t(577) = 3.73; p = .05] and rates of overweight or obesity [X^2^(1) = 4.08; p = .04] at baseline when compared to children who did not have measures for all time points.

### Behavioral observation

#### Total outside food and beverage items per child per week

**Figure 3:**
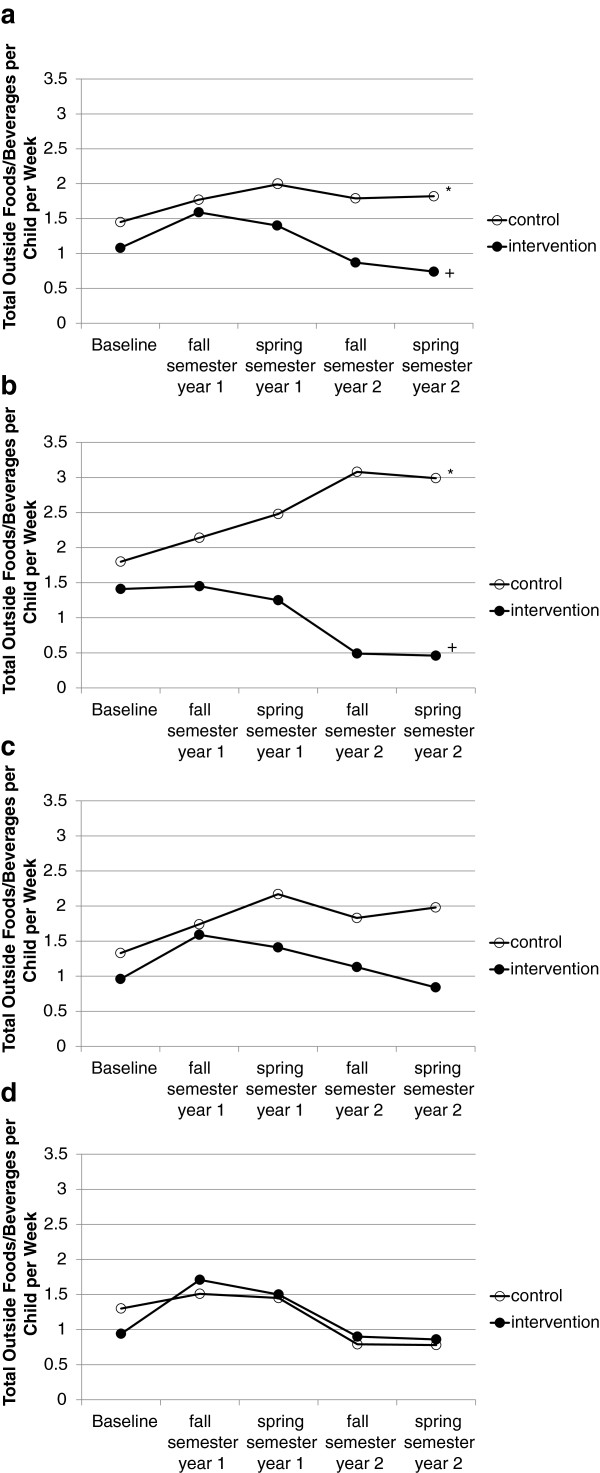
**a) Observed outside foods/beverages across all environments presented as average items per child per week in each semester of the study; b) Observed outside foods/beverages in the morning snack recess/playground environment presented as average items per child per week in each semester of the study; c) Observed outside foods/beverages in the classroom/school-wide events environment presented as average items per child per week in each semester of the study; d) Observed outside foods/beverages in the lunch/cafeteria environment presented as average items per child per week in each semester of the study.** *Significantly higher than baseline (p < .05); †Significantly lower than baseline (p < .05).

Findings for total outside foods and beverages observed per child per week are shown in Figures 3a. – 3d. There were no baseline differences between control and intervention schools for these measures. There was a significant group by time interaction [F(4,13) = 3.43; p = .04], such that intervention school outside food and beverage items per child per week decreased over time (p = .005) while these items in control schools increased over time (p = .04). This effect varied by school environment [F(8,26) = 2.77; p = .02], primarily determined by the morning snack recess/playground environment where outside foods/beverages in control schools increased (p < .001) and intervention schools decreased (p = .02). There were no differences between groups in outside foods/beverages for the classroom/school-wide events or school lunch/cafeteria environments. Outside foods/beverages in the lunch/cafeteria environment increased and then decreased over time for both groups (p < .01).

#### Unhealthy and healthy food and beverage items per child per week

Findings for unhealthy and healthy foods and beverages are shown in Table 3. There was a significant interaction of group by time for outside unhealthy food items [F(4,13) = 4.96; p = .01], such that the outside unhealthy food items on intervention school campuses decreased over time (p < .001) while these items increased over time in control schools (p = .02). This effect varied by school environment [F(8,26) = 2.76; p = .02], primarily determined by changes in the morning snack recess/playground environment where outside unhealthy foods in control schools increased (p = .01) and decreased in intervention schools (p = .01). There were no differences between groups for the classroom/school-wide events environment (although control schools had a marginal increase p = .06). Outside unhealthy food items in the lunch/cafeteria environment increased and then decreased over time for both groups (< .001). For outside unhealthy drink items, there was a significant group by time interaction [F(4,13) = 4.83; p = .01], such that unhealthy drink items on intervention school campuses decreased over time (p = .015) and control schools did not change. This effect did not vary significantly across school environments.

**Table 3:** Results for changes in outside foods/beverages on school campuses

	**Control Schools**			**Intervention Schools**		
	***Baseline***	***Year 1***	***Year 2***	***Baseline***	***Year 1***	***Year 2***
**Outside Unhealthy Foods**						
**Overall**	.67 ± .31	1.03 ± .38	.89 ± .55*	.47 ± .22	.71 ± .24	.32 ± .30^†^
**Recess**	.97 ± .30	1.26 ± .08	1.38 ± .18*	.68 ± .37	.67 ± .42	.16 ± .18^†^
**Classroom**	.66 ± .29	1.25 ± .36	1.13 ± .42	.44 ± .05	.79 ± .18	.49 ± .44
**Lunch**	.47 ± .19	.64 ± .17*	.29 ± .11^†^	.34 ± .05	.66 ± .18*	.25 ± .09^†^
**Outside Unhealthy Beverages**						
**Overall**	.32 ± .12	.35 ± .10	.28 ± .15	.26 ± .11	.23 ± .10	.09 ± .05^†^
**Recess**	.37 ± .10	.38 ± .05	.48 ± .04	.24 ± .14	.16 ± .12	.06 ± .05
**Classroom**	.21 ± .09	.32 ± .15	.25 ± .11	.19 ± .11	.21 ± .09	.09 ± .04
**Lunch**	.41 ± .08	.37 ± .08	.17 ± .04	.34 ± .06	.29 ± .07	.12 ± .05
**Outside Healthy Foods**						
**Overall**	.26 ± .09	.35 ± .16	.37 ± .28	.20 ± .11	,24 ± .11	.17 ± .11^†^
**Recess**	.33 ± .07	.57 ± .06	.74 ± .20	.33 ± .14	.25 ± .12	.16 ± .16^†^
**Classroom**	.26 ± .13	.29 ± .10	.33 ± .09	.16 ± .05	.17 ± .04	.14 ± .07
**Lunch**	.22 ± .04	.25 ± .07	.12 ± .04^†^	.14 ± .05	.31 ± .14	.21 ± .11*
**Outside Healthy Beverages**						
**Overall**	.12 ± .05	.19 ± .06	.16 ± .08	.10 ± .08	.14 ± .07	.09 ± .07
**Recess**	.07 ± .01	.19 ± .03	.25 ± .09	.08 ± .07	.10 ± .10	.06 ± .07
**Classroom**	.12 ± .05	.21 ± .09	.15 ± .07	.11 ± .12	.14 ± .06	.06 ± .02
**Lunch**	.15 ± .04	.16 ± .03	.11 ± .03	.09 ± .08	.17 ± .07	.15 ± .06

*Significant increase relative to baseline (p < .05); ^†^Significant decrease relative to baseline (p < .05).

Data are presented as items observed per child per week in the spring semester of each year.

There was a significant interaction of group by time for outside healthy food items [F(4,13) = 4.74; p = .01], such that outside healthy food items on intervention school campuses decreased (p = .03) and control school items did not change over time. This effect varied by school environment [F(8,26) = 4.38; p = .002]. In the morning snack recess/playground environment, outside healthy food items in control schools did not change, while intervention schools decreased (p = .02). The lunch/cafeteria environment showed the opposite effect with outside healthy foods increasing in intervention schools (p = .02) and decreasing in control schools (p = .02). There was no difference between groups for the classroom/school-wide event environment. There was no interaction of group or environment with time for observed outside healthy drink items. Over time, outside healthy drink items increased in all environments and schools until the last semester where there was a decrease [F(4,13) = 7.61 p = .002].

### Rates of overweight and obesity

Changes in rates of obesity (BMI ≤ 95^th^%) for intervention school children (28% baseline, 27% year 1, 30% year 2) were similar to those seen for control school children (22% baseline, 22% year 1, 25% year 2). There were also no intervention effects over time for rates of overweight or obesity (BMI ≤ 85^th^%) or BMI Z scores. Regardless of group or gender, all children increased significantly in BMI Z scores over time (p < .001).

## Discussion

As hypothesized using an implementation-focused EBPH approach to change nutrition environments and policies significantly decreased outside foods and beverages on campuses. The change was primary seen for unhealthy foods and beverages, although healthy foods were also reduced in the morning snack recess/playground environment. Conversely, healthy food items increased during lunch in intervention schools only. These intervention effects were likely a result of changing the following organizational policies and practices: substituting food/beverage rewards for nonfood/beverage rewards in the classroom, nutrition services catering healthy school-wide events and classroom celebrations, fundraising with healthy foods and beverages and nonfood activities such as “Jog-A-Thons”, adding a nutrition services prepared fruit snack at recess and not allowing outside foods and beverages in this environment, notifications sent home to parents about allowed healthy foods and beverages on campuses, school staff not consuming unhealthy foods and beverages in their classrooms, principles working to support teachers in turning parent unhealthy foods and beverages away when brought to campuses, and adding fruits and vegetables to school lunch entrees.

We also hypothesized that these nutrition environment and policy changes would result in significant differences between control and intervention school rates of obesity. This hypothesis was not supported as there were no changes in obesity rates across time in either control and intervention schools and BMI Z scores increased significantly over time for both intervention and control schools. There may be many reasons for this. Although we had a two-year intervention period, the implementation protocol took a full year to conduct before we had a set of intervention activities that could be enacted in all intervention schools. Thus the full intervention effect was only after one year of implementation. Future studies using this EBPH approach should allow for a longer intervention period (at least three years) to assess the maximal impact of the change strategies on child obesity rates.

In addition, we only targeted nutritional practices for change. The importance of adding PA to changes in dietary practices for sustained weight loss is well documented [47]. In our previous work with the El Paso Coordinated Approach to Child Health (CATCH) program, [35] our impact was much greater on obesity rates in the same intervention period of two years. In the El Paso CATCH program, both nutritional and PA practices were targeted for change and the largest effects were found for increases in PA during PE. Future studies should combine nutrition and PA changes to maximize the impact of any EBPH approach on child obesity rates. Finally, although parents were targeted through policy changes to prevent their sending unhealthy foods and beverages to school, this may have had little impact on nutrition practices at home. Simply targeting the school nutrition environment may not be enough to combat poor nutrition at home, especially in low income families like those targeted by Healthy ONES. Future studies should incorporate methods to study the dissemination of wellness policies to families and their implementation of good nutrition and PA outside of school settings.

The Healthy ONES intervention model has the potential to contribute significantly to the public health efforts to prevent obesity in children. Healthy ONES differs substantially from previous, more traditional EBM approaches for school health in that it followed an EBPH approach that engaged the school and district stakeholders in program planning and evaluation to address some of the organizational issues that could affect intervention effectiveness (such as financial concerns, labor issues, staff behavior, parental concerns, etc.). The Healthy ONES protocol for implementing school health change builds capacity within schools to make change and may increase the likelihood that schools will be able to sustain these changes. In addition, the Healthy ONES approach is relatively low cost compared to other interventions in the literature that required curriculum, staff training, and specialized instructors such as PE teachers. Healthy ONES provided a process for implementing environment and policy change with existing staff and required substitution rather than addition of activities. For example, instead of cakes for raffles to raise money the schools used prizes that cost the same amount as cakes. Instead of food-based fundraising, school instituted activity-based fundraisers such as “Jog-A-Thons” in which they made as much or more money than the food-based events.

Although Healthy ONES is the only school intervention to date that has used the IHI rapid improvement process for implementing health policy and environment change in public schools, there have been recent successful applications of some of the core principles in the Healthy ONES model, such as the use of participatory principles in making change and the adaptation of evidence-based interventions to address organizational and community concerns. For example, Pate and colleagues recently demonstrated an increase in PA among high school girls using a participatory approach [48] and Hoelscher and colleagues found better outcomes for child obesity by adding community involvement to their standard CATCH intervention [49]. In addition, our previous work with El Paso CATCH and Healthy Places emphasized sustainability by working within each system to build the capacity of existing staff to implement policy and environmental change [9,10,34,35].

Our past work with after-school programs has also shown success with building staff capacity to implement healthy environmental practice following a continuous training, quality improvement model [50]. Furthermore, Wiecha and colleagues [51] recently demonstrated that learning collaboratives following the IHI model are a promising tool for embedding health promotion practices in community-based after-school programs.

Perhaps the most striking evidence for the impact of capacity building and participatory-based approaches on obesity outcomes comes from two recent school health interventions, the School Nutrition Policy Initiative (SNPI) study [32] and the Healthy Living Cambridge Kids (HLCK) study [52]. The SNPI study did not explicitly state that they used CBPR, however, many of the elements of their intervention followed this approach. For example, they spent one year developing the intervention with the schools and communities in which they intervened. Staff development was a key component of the intervention with extensive training not only in the curriculum that was used, but in general concepts of nutrition and PA. This approach resulted in a marked reduction in obesity rates, especially for African American children, when compared to children in control schools.

The HLCK study was much more explicit in its use of CBPR and made concerted efforts to integrate the community into intervention strategies for school children. Their approach was similar to Healthy ONES in that they used the ecological models theoretical framework [31] to create strategies for change but spent many years in formative development and pilot testing of the actual intervention activities before they were implemented. They reported small, significant decreases in child obesity rates, although there was no control group to gauge the effect of time and secular trends.

There were a number of limitations with the Healthy ONES study design. One was the limited reach of the program (51%) primarily due to the low response rates for consent. The school district was low income as evidenced by 100% of children having free and reduced lunch rates. Different outreach methods may be necessary to insure higher participation rates in research studies for low income populations [53]. This low response rate may have also affected the power to detect differences between groups over time. We estimated that we would need 450 students at the end of the three year study and had slightly less than this at 424. One positive element of the study design was that the children for whom we had all height and weight measures were more obese than those who were lost to follow-up insuring that the children who most needed the intervention received the most exposure. Finally, measurement staff were not blinded to conditions. Funding limitations precluded our use of separate intervention and measurement staff. This may have positively biased the observational findings although every effort was made during training to emphasize the effect of bias on measurement.

## Conclusion

The Healthy ONES model for school nutrition policy and environmental change addresses many of the limitations of previous school interventions by using an EBPH approach focusing on processes of implementation and stakeholder participation. Healthy ONES *did not* focus only on achieving isolated policy and physical environmental changes such as removing vending and “junk” food sale opportunities, or simply changing meals offered by district nutrition services. The focus was on changing the organizational policies and practices of nutrition services, school staff, teachers, parents and students to improve the nutrition environment as well as implementing site-based strategies to assist schools in enforcing their existing federally-mandated wellness policy. Healthy ONES results suggest that the IHI rapid improvement process model for implementation can be used successfully in a variety of school settings to change nutrition policies and environments. EBPH community-driven process interventions, focusing on implementation, may be more likely to be disseminated and have greater effects across contexts because adaptation of the intervention is part of the intended intervention strategy.

## Competing interests

The authors declare that they have no competing interests.

## Authors’ contributions

KJC wrote the manuscript, conducted and interpreted the analyses, and served as the lead investigator on the study. MS, SLC, MEP wrote the manuscript and oversaw the implementation of the study. DAD wrote the manuscript and contributed to the analyses and its interpretation. All authors read and approved the final manuscript.
